# Hemolysis and cardiopulmonary bypass: meta-analysis and systematic review of contributing factors

**DOI:** 10.1186/s13019-023-02406-y

**Published:** 2023-10-13

**Authors:** Yudo P. Bhirowo, Yusuf K. Raksawardana, Budi Y. Setianto, Sudadi Sudadi, Tommy N. Tandean, Alfia F. Zaharo, Irhash F. Ramsi, Hening T. Kusumawardani, Teguh Triyono

**Affiliations:** 1 Department of Anesthesiology and Intensive Care, Faculty of Medicine, Public Health and Nursing, Universitas Gadjah Mada, Dr. Sardjito General Hospital, Jl. Kesehatan No. 1, Sendowo, Sekip Utara, Depok District, Sleman Regency, Yogyakarta, 55281 Indonesia; 2 Faculty of Medicine, Public Health and Nursing, Universitas Gadjah Mada, Yogyakarta, Indonesia; 3 Department of Cardiology, Faculty of Medicine, Public Health and Nursing, Universitas Gadjah Mada, Dr. Sardjito General Hospital, Yogyakarta, Indonesia; 4 Department of Ophthalmology, Faculty of Medicine, Public Health and Nursing, Universitas Gadjah Mada, Dr. Sardjito General Hospital, Yogyakarta, Indonesia; 5 Department of Clinical Pathology and Laboratory Medicine, Faculty of Medicine, Public Health and Nursing, Universitas Gadjah Mada, Dr. Sardjito General Hospital, Yogyakarta, Indonesia

**Keywords:** Cardiac, Surgery, Cardiopulmonary, Bypass, Hemolysis

## Abstract

**Background:**

The use of cardiopulmonary bypass (CPB) is almost inevitable in cardiac surgery. However, it can cause complications, including hemolysis. Until now, there have not been any standards for reducing hemolysis from CPB. Therefore, this systematic review was conducted to determine the factors that increase or reduce hemolysis in the use of CPB.

**Methods:**

Keywords Earches (cardiac surgery AND cardiopulmonary bypass AND hemolysis) were done on PubMed databases and Cochrane CENTRAL from 1990—2021 for published randomized controlled trials (RCTs) that studied interventions on CPB, in cardiac surgery patients, and measured hemolysis as one of the outcomes. Studies involving patients with preoperative hematological disorders, prosthetic valves, preoperative use of intra-aortic balloon pumps and extracorporeal circulation, emergency and minimally invasive surgery are excluded

**Results:**

The search yielded 64 studies that met the inclusion criteria, which involved a total of 3,434 patients. The most common surgery was coronary revascularization (75%). Out of 64 studies, 33 divided into 7 analyses. Remaining 31 studies were synthesized qualitatively. Significant decreases were found in centrifugal vs roller pumps for PFHb (*p* = 0.0006) and Hp (*p* < 0.0001) outcomes, separated vs combined suctioned blood (*p* = 0.003), CPB alternatives vs conventional CPB (*p* < 0.0001), and mini extracorporeal circulation (MiniECC) vs conventional CPB for LDH (*p* = 0.0008). Significant increases were found in pulsatility (*p* = 0.03) and vacuum-assisted venous drainage (VAVD) vs gravity-assisted venous drainage (GAVD) (*p* = 0.002).

**Conclusion:**

The review shows that hemolysis could be caused by several factors and efforts have been made to reduce it, combining significant efforts could be beneficial. However, this review has limitations, such as heterogeneity due to no standards available for conducting CPB. Therefore, further research with standardized guidelines for CPB is needed to yield more comparable studies. Meta-analyses with more specific parameters should be done to minimize heterogeneity.

**Supplementary Information:**

The online version contains supplementary material available at 10.1186/s13019-023-02406-y.

## Introduction

Since its conception, the use of cardiopulmonary bypass (CPB) is mostly inevitable in cardiac surgery, especially open-heart surgery. However, the use of CPB creates some supraphysiologic conditions such as exposure to non-endothelial surfaces, exposure to air, positive and negative pressure, and shear stresses. These conditions can contribute to complications, one of which is hemolysis [1].

Hemolysis is defined as the rupture of a Red Blood Cell (RBC), releasing its content such as Hemoglobin and Lactate Dehydrogenase (LDH) into the plasma. Hemoglobin released to the plasma or Plasma Free Hemoglobin (PFHb) will then bind to the circulating Haptoglobins (hp), which are then metabolized in the liver. However, when the release of hemoglobin is above the concentration of hps in the plasma, PFHb will exert its deleterious effects causing further complications such as Acute Kidney Injury (AKI) [2].

Many efforts have been made to reduce the complications of CPB due to hemolysis. Generally, each isolated component has been tested for its hemolytic characteristic, and once it is considered non-hemolytic, it can be used accordingly. However, cellular damage can be inflicted by the way each component is composed and managed in the full CPB circuit [1].

Along with managing the components of the circuits, several techniques and medications have been studied for their effect on reducing or increasing hemolysis. For example, pulsatility has been shown to increase PFHb [3]. Meanwhile, the use of pentoxifylline can reduce the increase of PFHb [4].

Until now, no guidelines nor standards are available for preventing hemolysis as one of the complications in the use of CPB. Accordingly, we conducted this review and meta-analysis of Randomized Controlled Trials (RCTs) to assess the effect of multiple interventions done on CPB for hemolysis in cardiac surgery patients.

## Methods

This review is registered in PROSPERO under ID CRD42021240131 submitted on April 30^th^, 2021. Preferred Reporting Items for Systematic Reviews and Meta-Analyses (PRISMA) statement was used to guide the search and develop the flow diagram.

### Search strategy

Keyword searches (cardiac surgery AND cardiopulmonary bypass AND hemolysis) were done on PubMed databases (1990–2023) and Cochrane CENTRAL (1990–2023) for published RCTs that studied interventions on CPB in cardiac surgery patients and measured hemolysis (PFHb, hp, LDH, or index of hemolysis) as one of the outcomes [5].

### Eligibility criteria

We included all studies with the following criteria:

- P: Cardiac surgery patients; and
- I: Cardiopulmonary Bypass with any additional methods (devices, techniques, and drugs) and its alternatives
- C: Cardiopulmonary Bypass with standard care
- O: Hemolysis, as one of its outcomes, through PFHb, hp, LDH, or index of hemolysis.
- S: Randomized Controlled Trials;

We excluded studies involving patients with preoperative hematological disorders, prosthetic valves, preoperative intra-aortic balloon pumps, pre-operative use of extracorporeal circulation, emergency cardiac surgery, and minimally invasive cardiac surgery, ongoing uncompleted studies.

### Data extraction and critical appraisal

Eight independent reviewers screened the abstracts for inclusion and exclusion criteria and assessed the studies’ eligibility. If any difference in assessments was found, the study was further discussed until a consensus was reached.

A standardized data extraction form was completed in a non-blinded fashion. Discrepancies in extracted data were rectified through consensus. Data were extracted from manuscript graphics as necessary using WebPlotDigitizer 5.4. Numerical outcome data were transformed into a common unit of measure. Mathematical approximations for mean and standard deviation (SD) were performed for manuscripts reporting median, range, and standard error.

To assess the risk of bias in selected studies, at least two reviewers working independently determined the bias in the randomization process, bias from deviation from intended intervention, bias from missing outcome data, bias in the measurement of the outcome, and bias in the selection of the reported results using the risk of bias assessment 2.0 tool (RoB 2.0). Whenever assessments were different, the study was further assessed to reach a consensus. The risk was described for every item as ‘low risk’ if the information provided in the study was clear and complete, ‘high risk’ if there was no information about some of the items, or the information provided revealed a clear risk of bias, and ‘some concerns’ when the information provided is incomplete.

### Statistical analysis

Statistical analysis was performed using the Review Manager, version 5.4 software (The Cochrane Collaboration, Oxford, UK), with a random-effects model. The results are presented as mean difference (MD) with 95% confidence intervals (CIs) for continuous data. All results were checked for statistical heterogeneity presenting the among-study variance τ^2^ and the chi-squared test. Statistical significance was set at a *p*-value of < 0.1 for heterogeneity. Inconsistency was tested using the I^2^ statistic and it was considered significant when it was 40%.

## Results

**Fig. 1:**
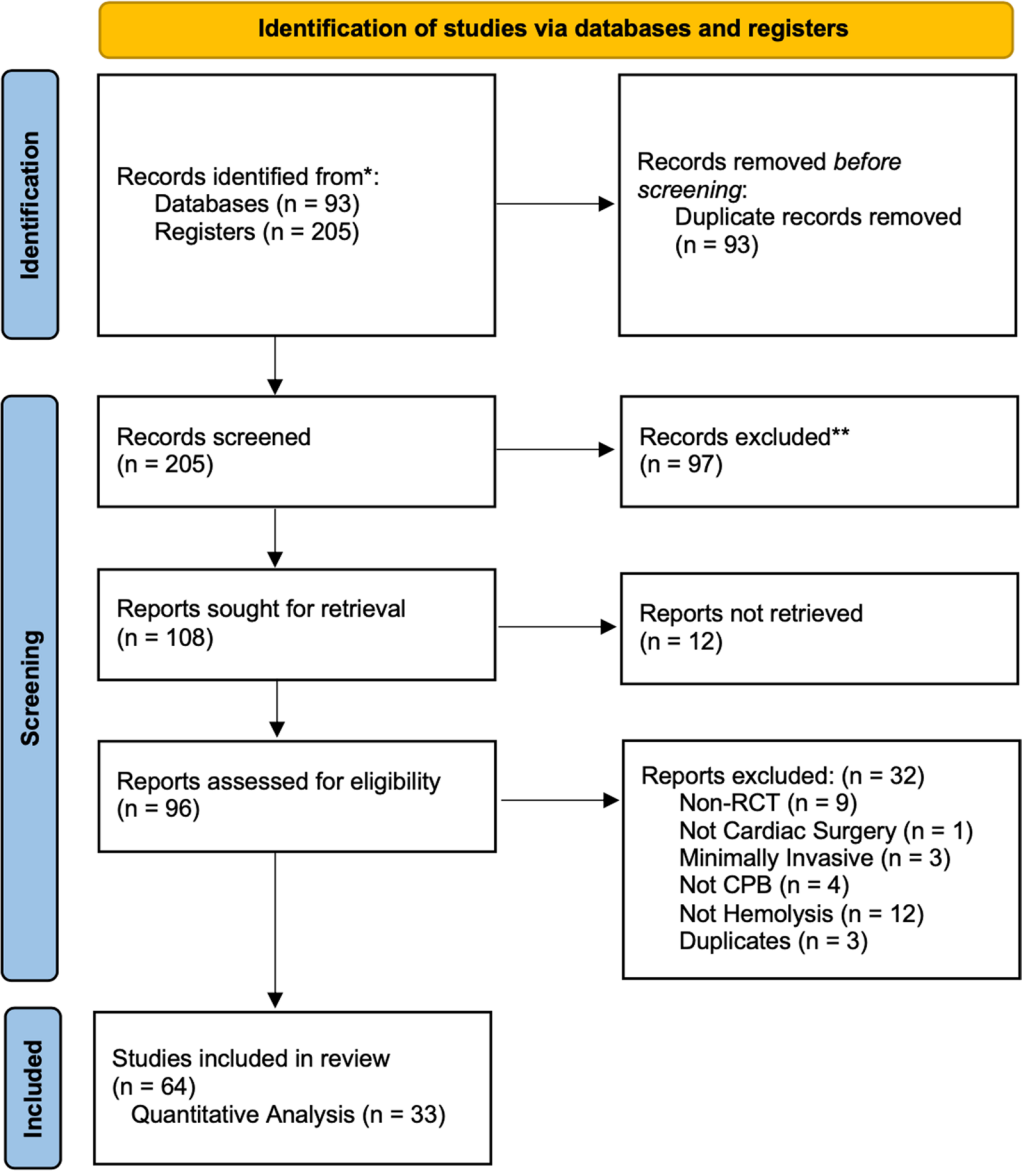
PRISMA flow chart summarizing study selection process. RCT indicates Randomized Controlled Trial; CPB, Cardiopulmonary Bypass

A total of 300 studies (95 from PubMed and 205 from Cochrane CENTRAL) were identified using the search criteria. Duplicate checking using Mendeley Reference Manager removed 93 studies. Abstract screening using *Abstrackr* (Brown.edu) identified 99 studies that did not fulfill the inclusion criteria and 108 studies that fulfilled the inclusion criteria and were sought for retrieval. There were 12 studies unavailable/not retrieved. There were 96 studies retrieved which were assessed for eligibility, 28 studies fulfilled the exclusion criteria, 3 duplicates were found, and 65 studies fulfilled the inclusion criteria (Fig. 1).

Out of the 65 included studies, only 33 studies could be analyzed quantitatively. Other studies were not analyzed quantitatively because of missing outcome data (n = 2), unconvertable units of measure (n = 2), high risk of bias (n = 1), and graph not available/eligible for extraction (n = 7), and incomparable studies (n = 17).

The 33 studies analyzed were divided into 7 main subgroups: types of pumps, types of oxygenators, venous drainage, coating, separated blood management, pulsatility, and alternatives to CPB.

### Study characteristics

All included studies were RCTs involving cardiac surgery patients analyzing different interventions on CPB and measuring hemolysis as one of their outcomes. A total of 3,384 cardiac surgery patients were enrolled with 2,589 undergoing coronary revascularization, 242 valvular heart surgery, 131 combined (coronary revascularization + valve surgery) procedures, 219 congenital heart surgery, and 203 other/unknown cardiac surgery.

Interventions varied regarding components of CPB such as types of pumps [6–18], types of oxygenators [19–22], types of cannulas [23], and types of suctions [24]; circuit management, such as separated reservoir [25–28], coating [29–37], venous drainage [38–40]; techniques, such as pulsatility [3, 41–44], retransfusion [45, 46], the use of cell saver [47–49], cardioplegia [50], priming [51], medications [4, 52–54], anesthesia [55]; and alternatives to CPB, such as MiniECC [56–64], off-pump coronary artery bypass (OPCAB) [58, 65], and Microaxial Intracardiac Pump (ICP) [66, 67].

All studies measured hemolysis as one of their outcomes either from PFHb levels, hp levels, LDH levels, or calculation of the index of hemolysis. There were 39 studies that measured only PFHb levels, 9 studies measured both PFHb and hp, 3 studies measured both PFHb and LDH, and 2 studies measured PFHb, hp, and LDH. Meanwhile, 3 studies measured only hp, 1 study measured both hp and LDH. There were 7 studies that measured only LDH, and only 3 studies calculated the hemolysis index. The parameters were measured either pre-, peri-, or postoperatively. Summary of findings tables are available (Tables 1, 2, 3, 4, 5).

**Table 1:** Summary of findings of published studies comparing plasma free hemoglobin (PFHb) and haptoglobin between centrifugal pumps and roller pumps

Pump, centrifugal compared to roller in cardiac surgery patients underwent CPB					
Outcomes	№ of participants (studies) Follow-up	Certainty of the evidence (GRADE)	Relative effect (95% CI)	Anticipated absolute effects	
				Risk with Roller	Risk difference with Pump, Centrifugal
Plasma Free Hemoglobin	168 (6 RCTs)	⨁⨁◯ Moderate	–	The mean plasma Free Hemoglobin was **0**	MD 22.27 lower (35.04 lower to 9.5 lower)
Haptoglobin	112 (3 RCTs)	⨁⨁⨁⨁ High	–	The mean haptoglobin was **0**	MD 24.81 higher (13.68 higher to 35.94 higher)

*The risk in the intervention group (and its 95% confidence interval) is based on the assumed risk in the comparison group and the relative effect of the intervention (and its 95% CI)

*CI* confidence interval, *MD* mean difference

*GRADE Working Group grades of evidence*

*High certainty*: we are very confident that the true effect lies close to that of the estimate of the effect

*Moderate certainty*: we are moderately confident in the effect estimate: the true effect is likely to be close to the estimate of the effect, but there is a possibility that it is substantially different

*Low certainty*: our confidence in the effect estimate is limited: the true effect may be substantially different from the estimate of the effect

*Very low certainty*: we have very little confidence in the effect estimate: the true effect is likely to be substantially different from the estimate of effect

**Table 2:** Summary of findings of published studies comparing plasma free hemoglobin (PFHb) and haptoglobin between vacuum assisted venous drainage (VAVD) and gravity assisted venous drainage (GAVD)

Vacuum assisted venous drainage compared to gravity assisted venous drainage in cardiac surgery patients underwent CPB					
Outcomes	№ of participants (studies) Follow-up	Certainty of the evidence (GRADE)	Relative effect (95% CI)	Anticipated absolute effects	
				Risk with placebo	Risk difference with Venous Drainage
Plasma Free Hemoglobin	170 (2 RCTs)	⨁⨁⨁⨁ High	–	The mean plasma Free Hemoglobin was 0	MD 5.37 higher (1.95 higher to 8.79 higher)

*The risk in the intervention group (and its 95% confidence interval) is based on the assumed risk in the comparison group and the relative effect of the intervention (and its 95% CI)

*CI* confidence interval, *MD* mean difference

*GRADE Working Group grades of evidence*

*High certainty*: we are very confident that the true effect lies close to that of the estimate of the effect

*Moderate certainty*: we are moderately confident in the effect estimate: the true effect is likely to be close to the estimate of the effect, but there is a possibility that it is substantially different

*Low certainty*: our confidence in the effect estimate is limited: the true effect may be substantially different from the estimate of the effect

*Very low certainty*: we have very little confidence in the effect estimate: the true effect is likely to be substantially different from the estimate of effect

**Table 3:** Summary of findings of published studies comparing plasma free hemoglobin (PFHb) between pulsatile and non-pulsatile

Pulsatile compared to non-pulsatile in cardiac surgery patients underwent CPB					
Outcomes	№ of participants (studies) Follow-up	Certainty of the evidence (GRADE)	Relative effect (95% CI)	Anticipated absolute effects	
				Risk with Non-Pulsatile	Risk difference with Pulsatile
Plasma Free Hemoglobin	204 (5 RCTs)	⨁⨁⨁⨁ High	–	The mean plasma Free Hemoglobin was **0**	MD 2.54 higher (0.3 higher to 4.78 higher)

*The risk in the intervention group (and its 95% confidence interval) is based on the assumed risk in the comparison group and the relative effect of the intervention (and its 95% CI)

*CI* confidence interval, *MD* mean difference

*GRADE Working Group grades of evidence*

*High certainty*: we are very confident that the true effect lies close to that of the estimate of the effect

*Moderate certainty*: we are moderately confident in the effect estimate: the true effect is likely to be close to the estimate of the effect, but there is a possibility that it is substantially different

*Low certainty*: our confidence in the effect estimate is limited: the true effect may be substantially different from the estimate of the effect

*Very low certainty*: we have very little confidence in the effect estimate: the true effect is likely to be substantially different from the estimate of effect

**Table 4:** Summary of findings of published studies comparing plasma free hemoglobin (PFHb) between different managements of suctioned blood

Suction blood management in cardiac surgery patients underwent CPB					
Outcomes	№ of participants (studies) Follow-up	Certainty of the evidence (GRADE)	Relative effect (95% CI)	Anticipated absolute effects	
				Risk with placebo	Risk difference with Suction Blood Management
Plasma Free Hemoglobin—Separated Reservoir	64 (3 RCTs)	⨁⨁◯◯ Low	–	The mean plasma Free Hemoglobin—Separated Reservoir was 0	MD 15.52 lower (28.29 lower to 2.75 lower)
Plasma Free Hemoglobin—Retransfusion	75 (2 RCTs)	⨁⨁⨁⨁ High	–	The mean plasma Free Hemoglobin—Retransfusion was 0	MD 14.69 lower (29.5 lower to 0.13 higher)

*The risk in the intervention group (and its 95% confidence interval) is based on the assumed risk in the comparison group and the relative effect of the intervention (and its 95% CI)

*CI* confidence interval, *MD* mean difference

*GRADE Working Group grades of evidence*

*High certainty*: we are very confident that the true effect lies close to that of the estimate of the effect

*Moderate certainty*: we are moderately confident in the effect estimate: the true effect is likely to be close to the estimate of the effect, but there is a possibility that it is substantially different

*Low certainty*: our confidence in the effect estimate is limited: the true effect may be substantially different from the estimate of the effect

*Very low certainty*: we have very little confidence in the effect estimate: the true effect is likely to be substantially different from the estimate of effect

**Table 5:** Summary of findings of published studies comparing plasma free hemoglobin (PFHb) between CPB and alternatives to CPB

Alternatives to CPB compared to CPB in cardiac surgery patients					
Outcomes	№ of participants (studies) Follow-up	Certainty of the evidence (GRADE)	Relative effect (95% CI)	Anticipated absolute effects	
				Risk with placebo	Risk difference with Alternative ECC
Plasma Free Hemoglobin—MiniECC	235 (5 RCTs)	⨁⨁⨁◯ Moderate	–	The mean plasma Free Hemoglobin—MiniECC was **0**	MD 20.66 lower (35.76 lower to 5.56 lower)
Plasma Free Hemoglobin—OPCAB	135 (3 RCTs)	⨁⨁⨁◯ Moderate	–	The mean plasma Free Hemoglobin—OPCAB was **0**	MD 25 lower (44.65 lower to 5.35 lower)

*The risk in the intervention group (and its 95% confidence interval) is based on the assumed risk in the comparison group and the relative effect of the intervention (and its 95% CI)

*CI* confidence interval, *MD* mean difference

GRADE Working Group grades of evidence

*High certainty*: we are very confident that the true effect lies close to that of the estimate of the effect

*Moderate certainty*: we are moderately confident in the effect estimate: the true effect is likely to be close to the estimate of the effect, but there is a possibility that it is substantially different

*Low certainty*: our confidence in the effect estimate is limited: the true effect may be substantially different from the estimate of the effect

*Very low certainty*: we have very little confidence in the effect estimate: the true effect is likely to be substantially different from the estimate of effect

### Risk of bias assessment

Most of the included studies have some concerning bias. Only 16 studies have a low-risk bias. The bias in the randomization process was assessed in 41 studies. One study has concerning bias due to deviation from the intended intervention. There was one study with a high risk of bias due to possible differences in study protocol between groups which could lead to differences in the outcome measured. Seven studies were assessed having a high risk of bias due to selective reporting of the outcomes. The studies’ characteristics are available in the Additional file 1.

### Quantitative data analysis

#### Types of pumps

**Fig. 2:**
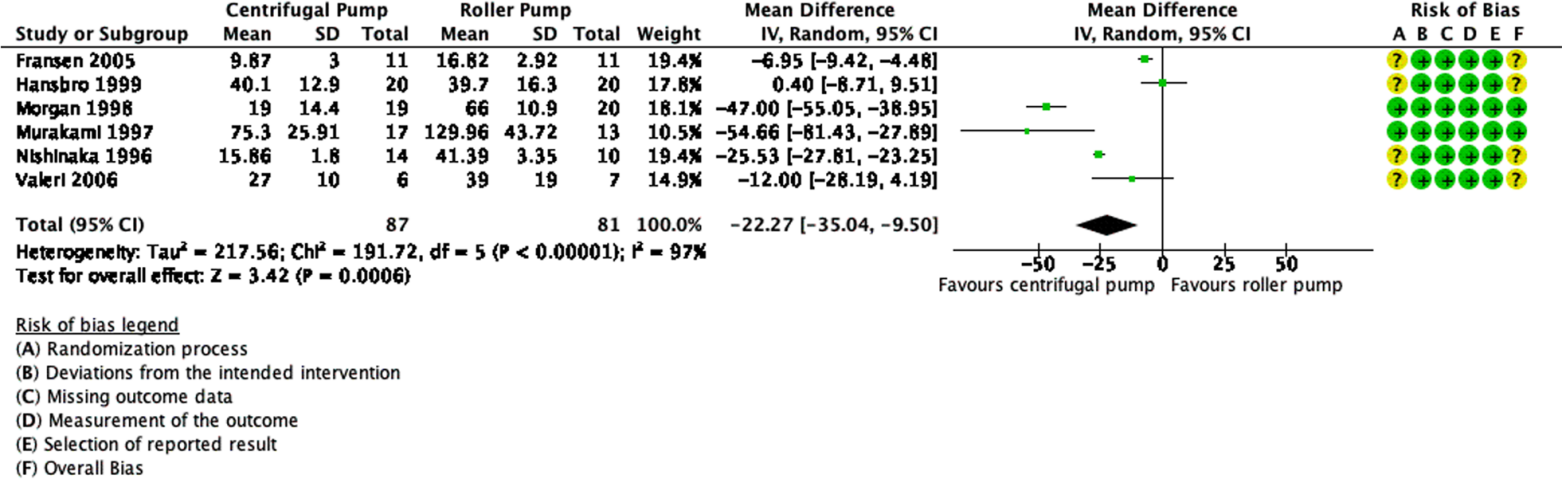
Forest plot of published studies comparing plasm free hemoglobin (PFHb) between centrifugal pumps and roller pumps using random effects analysis. Data presented as Mean Difference (MD) with 95% Confidence Interval (CI)

Figure 2 shows a total of 87 and 81 patients were analyzed in the centrifugal pump (CP) group and roller pump (RP) group for the outcome PFHb, respectively. Pooled estimates showed that there is a significant difference in PFHb levels between the two groups (*p* = 0.0006). The use of CP decreased the event of hemolysis through PFHb, as much as 22.27 mg/dL with 95% CI − 35.04 to − 9.50. There were significant heterogeneity and inconsistency (*p* < 0.00001, I^2^ = 97%).

**Fig. 3:**
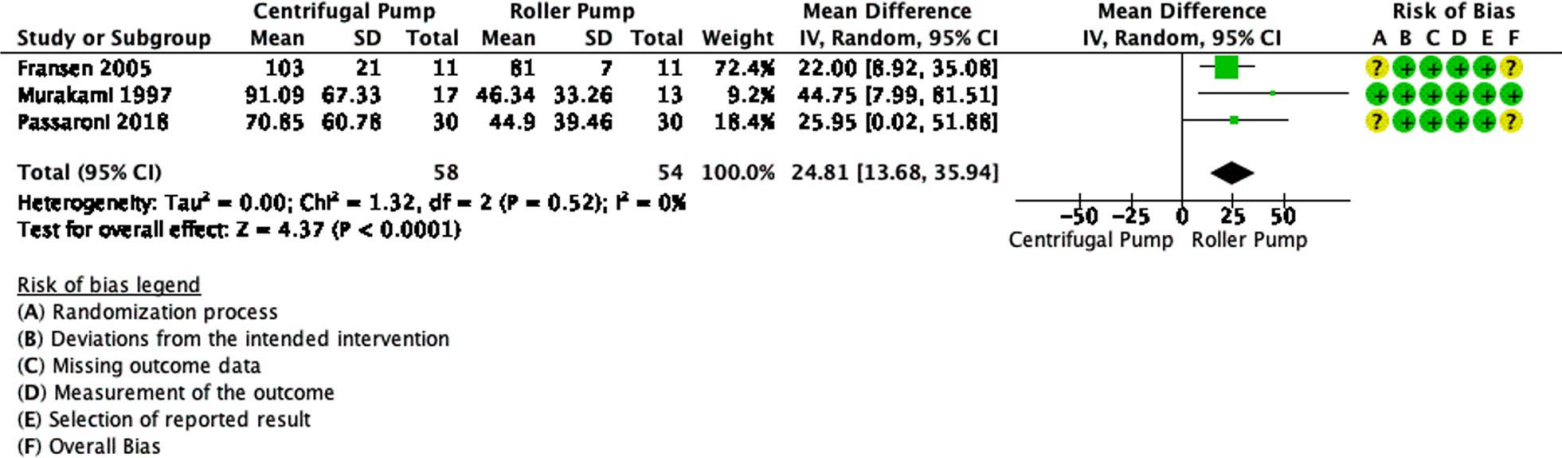
Forest plot of published studies comparing haptoglobin (hp) between centrifugal pumps and roller pumps using random effects analysis. Data presented as Mean Difference (MD) with 95% Confidence Interval (CI)

Figure 3 shows a total of 58 and 54 patients were analyzed in the CP group and RP group for the outcome of hp, respectively. Pooled estimates showed that there was a significant difference in PFHb levels between the two groups (*p* < 0.0001). The use of CP decreased the event of hemolysis based on higher hp concentrations by 24.81 mg/dL with 95% CI: 13.68 to 35.94. There were no significant statistical heterogeneity nor inconsistency (*p* = 0.52, I^2^ = 0%).

#### Venous drainage

**Fig. 4:**
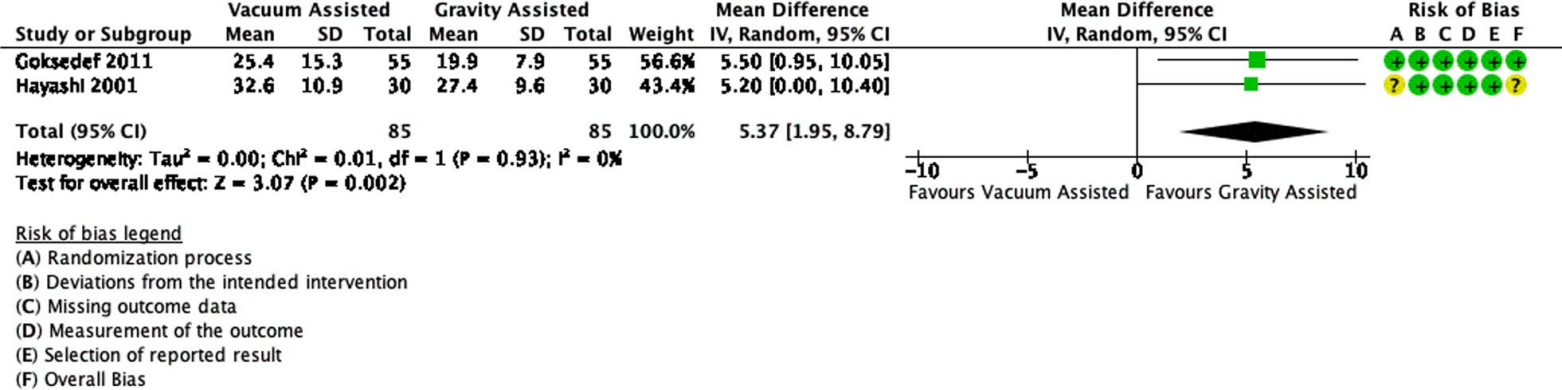
Forest plot of published studies comparing plasma free hemoglobin (PFHb) between vacuum-assisted venous drainage (VAVD) and gravity-assisted venous drainage (GAVD) using random effects analysis. Data presented as Mean Difference (MD) with 95% Confidence Interval (CI)

As shown in Fig. 4, the total number of patients both in vacuum-assisted venous drainage (VAVD) and gravity-assisted venous drainage (GAVD) groups was 85. Pooled estimates showed that there was a significant difference between both groups (*p* = 0.002). The use of VAVD increased the risk of hemolysis, through PFHb, as much as 5.37 mg/dL compared to GAVD with 95% CI:1.95 to 8.79. There were no significant statistical heterogeneity nor inconsistency (*p* = 0.93, I^2^ = 0%).

### Suction blood management

**Fig. 5:**
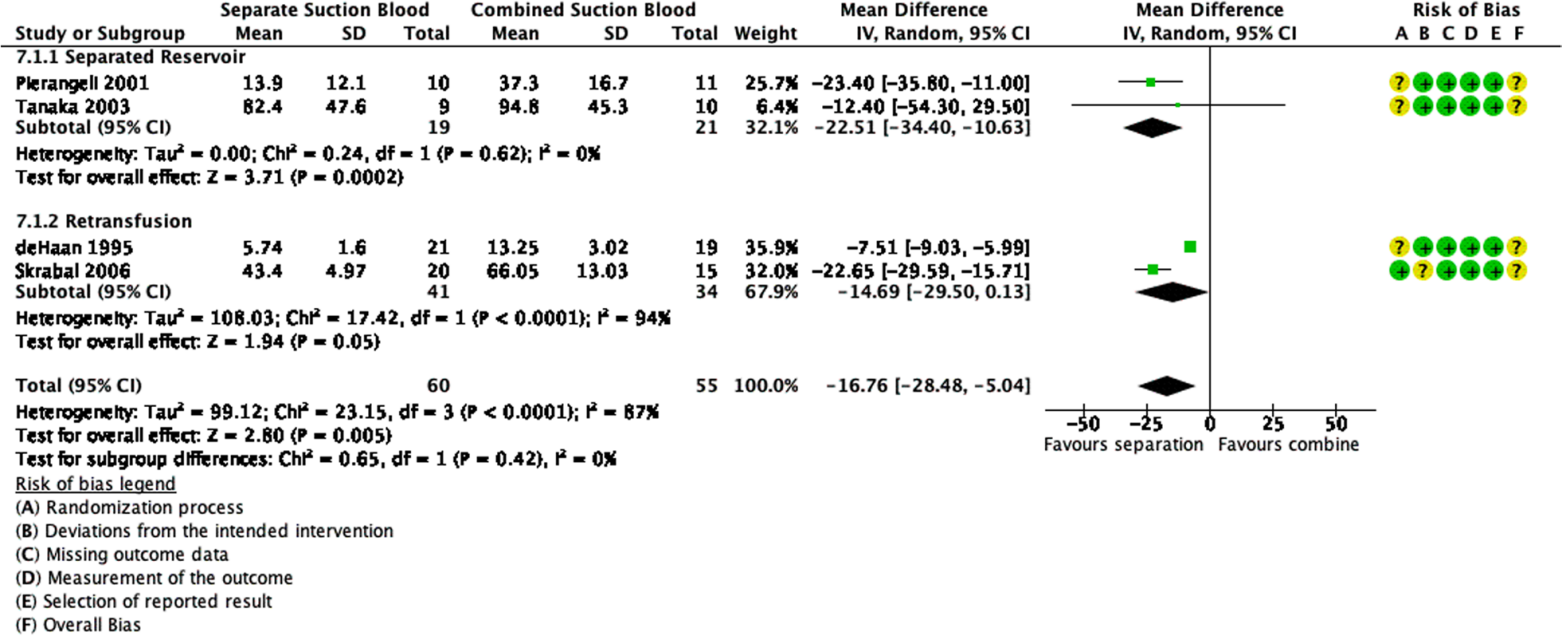
Forest plot of published studies comparing plasma free hemoglobin (PFHb) of suction blood management using random effect analysis. Subgroup analyses were d006Fne for separated reservoir and retransfusion vs retainment methods using random effects analysis. Data presented as Mean Difference (MD) with 95% Confidence Interval (CI)

Figure 5 shows that there were 72 patients in the separated suctioned blood group and 67 patients in the combined suctioned blood group. Pooled estimates showed that separating suctioned blood reduced the PFHb levels significantly, therefore hemolysis, as much as 16.76 mg/dL (95% CI − 28.48 to − 5.04, *p* = 0.005). There were significant statistical heterogeneity and inconsistency (*p* < 0.0001, I^2^ = 87%). However, there were no significant subgroup differences (*p* = 0.93, I^2^ = 0%).

*Separated Reservoir.* Figure 5 shows that a total of 31 patients were enrolled in a separated reservoir group and 33 patients were enrolled in a combined reservoir group. The pooled estimates showed that the use of separated reservoirs significantly decreased hemolysis, through PFHb levels, as much as 22.51 mg/dL (95% CI − 34.40 to − 10.63, *p* = 0.0002). There were no significant statistical heterogeneity nor inconsistency (*p* = 0.23, I^2^ = 32%).

*Retransfusion*. Figure 5 shows that 34 patients belonged to the retransfusion of suctioned blood group and 41 patients belonged to the retainment of suctioned blood group. Meta-analysis shows that there was no significant difference between the two groups, although retainment decreased hemolysis by PFHb levels as much as 14.69 mg/dL in comparison to retainment of suctioned blood (*p* = 0.05, 95% CI − 29.50 to 0.13). However, there were statistically significant heterogeneity and inconsistency (*p* < 0.00001, I^2^ = 94%) (Fig. 9).

### Pulsatility

**Fig. 6:**
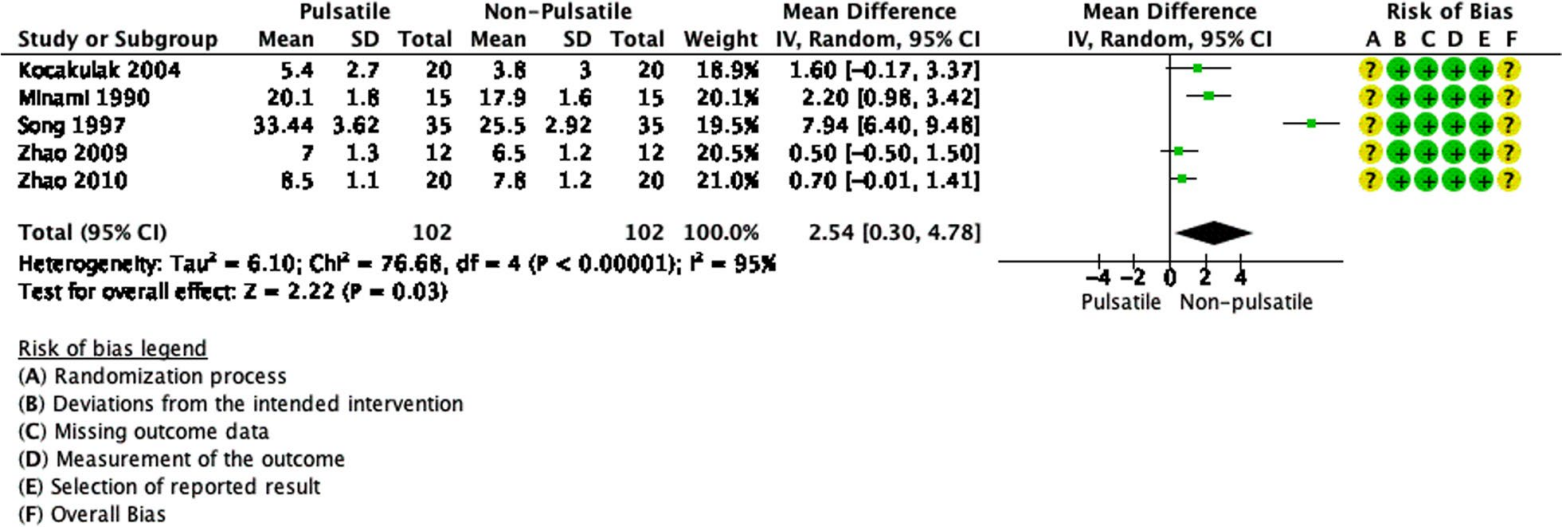
Forest plot of published studies comparing plasma free hemoglobin (PFHb) between pulsatile and non-pulsatile perfusion using random effects analysis. Data presented as Mean Difference (MD) with 95% Confidence Interval (CI)

Figure 6 shows that a total of 204 patients, divided into the pulsatile and non-pulsatile groups, were included in the analysis. There was a significant difference in hemolysis between pulsatile and non-pulsatile group (*p* = 0.03). Pulsatile perfusion increased the event of hemolysis from PFHb levels by as much as 2.54 mg/dL in comparison to non-pulsatile perfusion (95% CI 1.17 to 2.13). However, there were statistically significant heterogeneity and inconsistency (*p* < 0.00001, I^2^ = 95%).

### Alternatives to CPB (*MiniECC* and OPCAB)

**Fig. 7:**
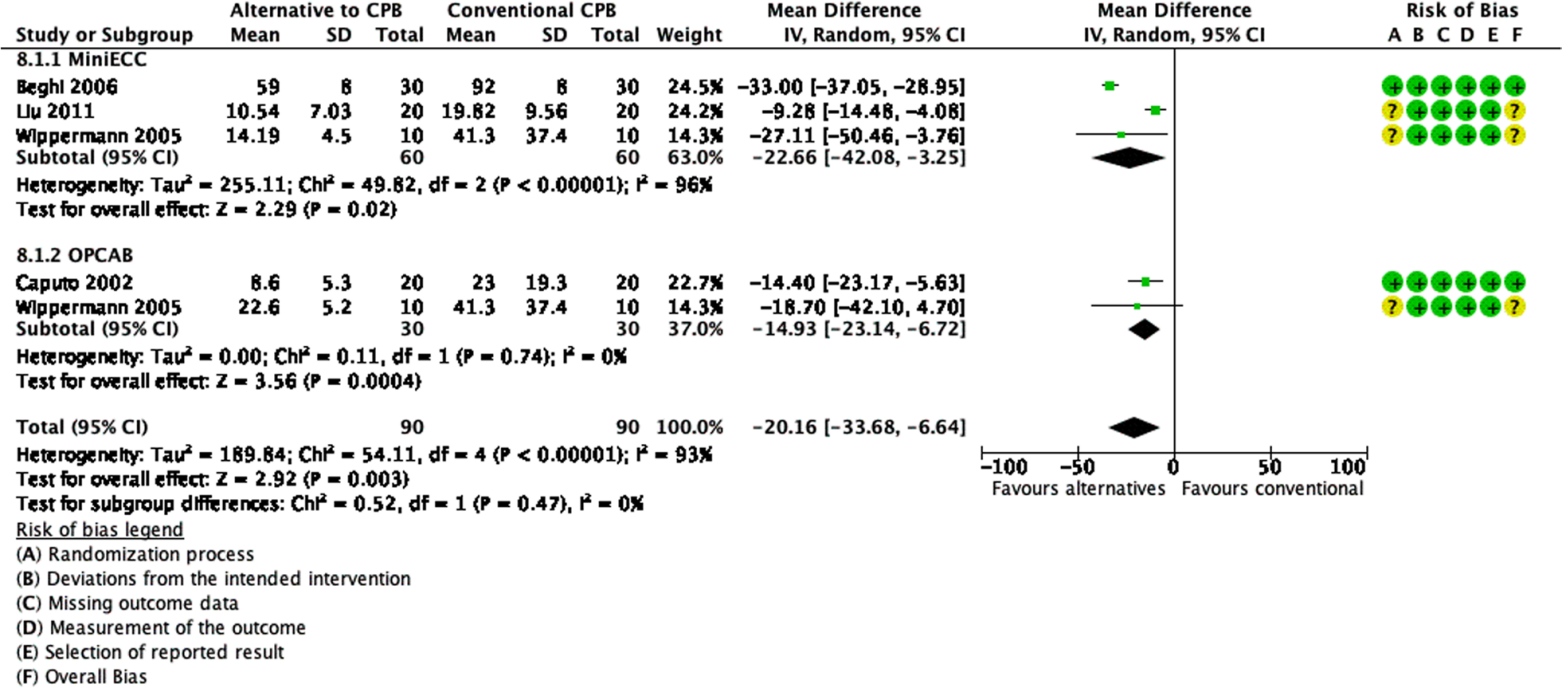
Forest plot of published studies comparing plasma free hemoglobin (PFHb) of CPB alternatives using random effect analysis. Subgroup analyses were done for mini extracorporeal circulation (MiniECC) and off-pump coronary artery bypass (OPCAB) using random effects analysis. Data presented as Mean Difference (MD) with 95% Confidence Interval (CI)

Figure 7 shows the pooled estimates from 182 patients in the alternative group and 188 patients in the CPB group. The use of alternatives to CPB significantly decreased hemolysis, by PFHb levels, as much as 20.16 mg/dL (95% CI − 33.68 to − 6.64, *p* = 0.003). There were statistically significant heterogeneity and inconsistency (*p* < 0.00001, I^2^ = 93%). There were no significant subgroup differences (*p* = 0.47, I^2^ = 0%).

*Mini ECC*. Figure 7 shows that a total of 106 and 109 patients were analyzed in the mini ECC and conventional CPB groups for the outcome PFHb, respectively. There was a significant difference between groups, in which, mini ECC decreased hemolysis, by PFHb level, as much as 22.66 mg/dL (*p* = 0.02, 95% CI − 42.08 to − 3.25). However, there were statistically significant heterogeneity and inconsistency (*p* < 0.00001, I^2^ = 96%).

**Fig. 8:**
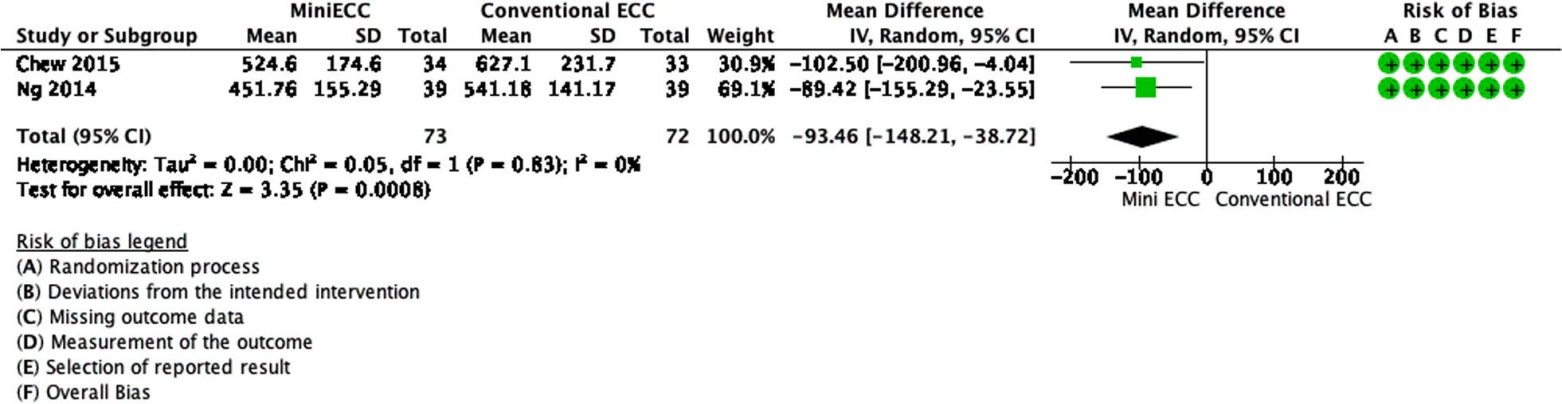
Forest plot of published studies comparing lactate dehydrogenase (LDH) between mini extracorporeal circulation (MiniECC) and conventional extracorporeal circulation (CECC) using random effects analysis. Data presented as Mean Difference (MD) with 95% Confidence Interval (CI)

Meanwhile, Fig. 8 shows that there were 73 and 72 patients divided into the mini CPB and conventional CPB groups for the outcome LDH, respectively. The pooled estimates showed that there was a significant difference between the two groups (*p* = 0.0008). Mini CPB decreased hemolysis, by LDH levels, as much as 93.46 U/L (95% CI − 148.21 to − 38.72). There were no significant statistical heterogeneity nor inconsistency (*p* = 0.83, I^2^ = 0%).

*Off-Pump Coronary Artery Bypass (OPCAB).* Figure 7 shows that there were 66 patients and 69 patients in the OPCAB and conventional CPB groups, respectively. Pooled estimates showed that OPCAB significantly decreases PFHb levels, therefore hemolysis, as much as 25 mg/dL (95% CI − 44.65 to − 5.35, *p* = 0.01). There were significant statistical heterogeneity and inconsistency (*p* < 0.0001, I^2^ = 90%).

## Discussion

Hemolysis is one of the concerning complications in the use of cardiopulmonary bypass (CPB) because it can lead to other further complications such as acute kidney injury (AKI) [26]. Our systematic review and meta-analysis aimed to assess the contributing factors of hemolysis as well as efforts to reduce it by synthesizing the results from all RCTs available from 1990 to 2021. A total of 64 studies met our inclusion criteria, and we were able to perform meta-analyses in some of the studies found (n = 33). A narrative synthesis was done in the remaining studies.

The findings of this review indicated that most of the interventions done in the studies did not show any significant improvement, in terms of hemolysis, in comparison to standard methods. However, most of the analyses found significant heterogeneity and inconsistency between groups. This finding could be caused by the fact that there are a variety of CPB designs and circuit settings, and the fact that there are no standard guidelines for conducting CPB, along with the differences in the populations studied. We incorporated both adult and pediatric patients, with a variety of types of cardiac surgery, in the analysis due to minimal evidence regarding CPB and hemolysis.

### Types of pumps

From our analysis, centrifugal pumps (CP) showed a significant reduction in hemolysis in terms of PFHb and Hp levels in comparison to roller pumps (RP). This finding, however, was not shown in the LDH levels. Meanwhile, from the narrative synthesis, conflicting findings were found. While two studies reported no significant difference in hemolysis, one study reported a significant difference.

For PFHb levels, several studies reported significant differences [8, 10–12] and other studies reported no significant differences [6, 7, 14, 16]. Hansbro et al. reported no difference between groups, which could be due to the relatively short duration of CPB time (84.1 ± 21.9 min in CP and 87.2 ± 19.5 in RP) [6]. This statement is supported by Andersen et al. who reported an average CPB time of 84 min [16]. Nishinaka et al. found a significant difference after 90 and 120 min of CPB [12]. Morgan et al. found a similar result in pediatric CPB with a mean time of more than 90 min [11]. Meanwhile, Murakami et al. and Fransen et al. found a significant difference in CPB time of fewer than 90 min. This difference, however, could be because of the controlled experiment setting in which in Murakami et al., there was a low negative pressure CPB and in Fransen et al., there was a separation of pericardial suctioned blood [8, 10]. Wheeldon et al. reported higher hemolysis in the CP group, however, this study also reported a high standard of error [14]. Overall, CP appeared to be superior than RP, in terms of PFHb levels, but only if the CPB duration is more than 90 min.

Alternatively, another systematic review by Saczkowski et al. did not find a significant difference in PFHb levels between CP and RP [68].

The reduction of haptoglobin (hp) was reported to be significant, between CP and RP, in 2 out of 3 studies [8, 10]. Passaroni et al. reported no significant between-group difference, but there were significant within-group differences between pre-CPB and post-CPB, both in the CP and RP groups [9]. This shows that CPB duration is a more important factor for hemolysis than types of pumps. Meanwhile, for the LDH levels, our analysis did not find any significant differences between CP and RP. There were 2 out of 3 included studies that did not find any significant differences [7, 13]. Meanwhile, Murakami et al. found a significant difference in post-CPB LDH levels [8]. These conflicting results could be because LDH is not a specific marker of erythrocyte injury. LDH also rises in other cellular injuries, which can happen in CPB.

Studies comparing different types of CPs did not find any significant difference in terms of hemolysis [17, 18]. Overall, it is safe to say that CPs remained superior to RPs, especially in CPB duration of more than 90 min.

### Types of oxygenators

Other components that spark interest are oxygenators. Oxygenators have a high blood contact rate and there are various types of oxygenators. Differences in materials and design can contribute to a certain degree of blood damage [19]. Our analysis compared two different types of oxygenators, the hollow-fiber membrane (HFMO) and membrane oxygenators (MO). We found that HFMO induces more hemolysis than MO, from higher PFHb levels. However, the difference was not significant. The studies included in the analysis also had conflicting results. Benedetti et al. found superiority in MO in terms of PFHb levels [19]. Meanwhile, Stammers et al. found HFMO caused less hemolysis than MO [20]. Benedetti et al. also compared other types of oxygenators including Bubble Oxygenators (BO) and Hybrid Oxygenators (HO), and found that BO causes the most hemolysis and MO causes the least, with HFMO and HO in the intermediate level. However, in their arguments, the cause of hemolysis could be because of other factors along the ECC circuit [19]. Additionally, Stammers et al. described that in HFMO, there is less transmembrane pressure drop which explained the less hemolysis in HFMO [20]. However, Simon et al. found no difference in hemolysis despite a significant difference in pressure drop between two HFMOs [21]. Chukwuemeka et al. compared two different types of oxygenators with two different priming volumes and found no significant difference in terms of hp levels [22]. To conclude, oxygenators may play a role in blood damage and different types of oxygenators did not differ significantly. However, hemolysis still occurs. This could be caused by other components of CPB circuits.

### Venous drainage

Our analysis shows that the use of VAVD increases PFHb levels in comparison to GAVD. However, individual studies show conflicting results. Goksedef et al. found a significant difference at 2 h and 24 h postoperative [39]. Meanwhile, Hayashi et al. and Bevilacqua et al. did not find any difference [38, 40]. Goksedef et al. stated that the significant difference was between VAVD at 80 mmHg and VAVD at 40 mmHg or GAVD, indicating that pressure affects hemolysis [39]. Meanwhile, Hayashi et al. set the pressure of VAVD to be 30 mmHg, while Bevilacqua et al. determined it as 29 ± 8.9 mmHg [38, 40] Overall, the use of VAVD could induce hemolysis at greater pressure. However, according to Hayashi et al., the use of VAVD could reduce the priming volume thus reducing hemodilution [38]. Less hemodilution could be beneficial in minimizing blood damage and subsequent hemolysis.

## Suction blood management

Cardiotomy suction (CS) is known to be the largest source of blood damage due to the amount of air entrapment causing turbulence and high shear stress to red blood cells [1]. Jegger et al. found that hemolysis, from LDH and PFHb levels, were dependent on CS, CPB time, and type of surgery. The same study tested a novel device capable of minimizing blood-air mixing through an optic sensor that aspirates only when blood is detected. It was found that PFHb and LDH values were significantly lower in the SS group than in CS. The findings were also lower in the CABG surgery vs. valve surgery, where the valve surgery requires a larger field of surgery, thus increasing blood-air contact and use of CS. In conclusion, one of the major sources of hemolysis is blood-air contact, which happens with the use of CS. Therefore, reducing blood-air contact could result in less hemolysis, such as seen in the use of SS devices [24].

Hemolysis, caused by CS, could be reduced by separating the suctioned blood. There are several methods to separate suction blood, such as separating reservoirs and retainment of suctioned blood. Our analysis found that separating suctioned blood, overall, significantly reduced PFHb levels, thus reducing hemolysis. Subgroup analysis of separate reservoirs also found a significant reduction in PFHb levels. However, individual studies found conflicting results. The studies conducted by Tanaka et al. and Gunaydin et al. did not find any significant difference between open circuits (combined reservoir) and closed circuits (separated reservoir) [25, 27]. Meanwhile, Pierangelli et al. and Nasso et al. found a significant difference between the two groups [26, 28]. Tanaka et al. mentioned the use of a cell saver, which could explain their not significant results [25]. Overall, separating reservoirs for suctioned blood seemed to be more beneficial than combined reservoirs in reducing hemolysis. Reduction of hemolysis would be better if combined with other methods of suctioned blood management.

Retaining suctioned blood entirely could also be beneficial in reducing hemolysis. Our analysis showed that retainment does reduce hemolysis more than retransfusion. Although the differences found were not significant. Both individual studies show significant differences when compared between retransfusion and retainment. However, de Haan et al. mentioned that PFHb levels increased proportionally after retransfusion. This result shows further hemolysis does not happen after retransfusion [46]. But overall, retransfusion of suctioned blood should be avoided as much as possible. The use of a cell-saver could be used when needed, such as in surgery with significant blood loss.

There are several techniques of cell-saving. We found studies describing plateletpheresis (PRP), cell-washing, and hemadsorption. Boey et al. showed that pre-operative plateletpheresis increases hemolysis significantly. This could be due to the fragility of RBC from the PRP procedure [47]. Another method, studied by Walpoth et al., is cell-washing using a continuous auto-transfusion system (CATS). They found a significant reduction in PFHb levels in the CATS group [49]. Gleason et al. also found a significant reduction of PFHb levels through CytoSorb devices. CytoSorb employs the hemadsorption method of cell-saving [48]. Therefore, several cell-saving devices, such as CATS and CytoSorb*,* are beneficial in reducing PFHb levels, thus reducing the damaging effect of hemolysis.

Overall, the use of CS remains one of the major sources of hemolysis and should be paired with other management such as separation and retainment of the suctioned blood, to reduce the effect of hemolysis in general. Cell-washing could be an alternative when separation and retainment are not possible. Cell-washing methods vary but we found CATS and CytoSorb to be beneficial.

### Types of cannulas

Venous cannula, often, is large enough to keep the flow laminar with minimal turbulence [1]. Jegger et al. also did not find any significant difference in PFHb and LDH levels when comparing traditional venous cannula and a novel self-expanding SmartCannula (SC). SC is shown to have a superior flow characteristic to the regular two-stage venous cannula, in such pressure gradients were lower in SC. However, the difference did not affect hemolysis [23].

### Coating

The coating is used in CPB to mimic natural endothelium and decrease the effect of blood and material contact. Two major types of coating are generally used in CPB, bioactive (Heparin, NO) and passive (albumin, PEO, and phosphorylcholine) [37]. Our review found studies discussing heparin coating, phosphorylcholine coating (Pc), and PMEA coating. Analysis of studies comparing coated circuits and uncoated circuits, with heparin or phosphorylcholine, show a reduction in PFHb levels. However, the reduction was not significant. Similar findings were found in a subgroup analysis of the heparin-coated circuit and phosphorylcholine-coated circuit. Individual studies also reported no significant difference between groups [29, 30, 35]. Sellevold et al. stated that the increased PFHb levels were more likely to be due to autotransfusion. However, the use of heparin coating resulted in less heparinization of the patient’s blood, creating potential benefits against the harmful effect of heparin [30]. Moen et al. found that the use of heparin coating reduces terminal complement complex (TCC) significantly. TCC reflects the component of ‘bystander lytic attack’ on cells, therefore suggesting less red blood cells ‘attack’ with heparin coating [35].

Similar results were found in Pc coating, as well as findings in individual studies [31, 32, 36]. De Somer et al. stated that major hemolysis is caused by aspiration of blood from non-vascular cavities, such as suction. Hemolysis from blood contact to non-endothelial surfaces seems to be negligible in short-term surgery and can be compensated by rapid elimination of hp and hemopexin [31].

When comparing Pc coating and other coatings, no significant differences were found. Individual studies comparing Pc coating and heparin coating found no significant difference in PFHb levels [37]. However, LDH levels were significantly increased in Pc coating, 24 h postoperatively. The difference was caused by a longer duration in the Pc coating group (128 vs 101 min) [33]. LDH levels were similar when compared to PMEA coating. It should be noted that TCC significantly increased in the Pc coating group when compared to PMEA, but the results were similar when compared to heparin coating. However, the clinical significance is yet to be known [34].

Overall, the result suggests that blood and non-endothelial contact only induce minor hemolysis that can be compensated by the haptoglobin-hemopexin clearance system. Types of coating did not differ significantly in terms of inducing hemolysis.

### Pulsatility

Pulsatility is still a controversial discussion, in the debate concerning whether pulsatile or continuous perfusion is superior to the other. In terms of hemolysis, however, our analysis found a significant increase in PFHb levels in the pulsatile perfusion method. However, individual results are conflicting. Minami et al. did not find any significant difference [41]. This is supported by the results of Kocakulak et al [3]. Another study conducted by Song et al. did find a significant difference [42]. Similar findings were found in studies by Zhao et al [43, 44]. Although most of the findings still favor continuous perfusion to be superior. Shear stress in pulsatile perfusion is thought to be higher, therefore causing more hemolysis. However, Kocakulak et al. stated with the appropriate choice of materials, hemolysis could be compensated [3]. Additionally, Song et al. stated that hemolysis induced by pulsatile perfusion is still within the normal range [42]. Overall, continuous perfusion still produces less hemolysis than pulsatile perfusion and should be chosen to reduce hemolysis in CPB.

### Cardioplegia

Blood cardioplegia is shown to have a better effect on hemolysis than cardioplegia. However, the difference was not significant. Rinne et al. stated that the increase of PFHb in both groups could be due to other factors such as the use of bubble oxygenators [50].

### Priming

Most priming solutions will cause hemodilution and a decrease in oncotic pressure. Hemodilution is known to be a contributing factor to hemolysis, increasing the mechanical fragility of RBCs. Therefore, the need for better fluid for priming is needed. Barbu et al. found that dextran-based prime (hyper-oncotic fluid) is superior, in terms of hemolysis, to crystalloid based-primer. Although the mechanism is not fully understood, it is thought that dextran-based primer reduces the effects of shear stress on red blood cells [51].

### Medication

Several medications are known to have some effect in modulating hemolysis or detrimental effects of hemolysis. For example, pentoxifylline (PTX) is known to have hemorheological properties, increasing RBC deformability and decreasing blood viscosity [4]. Nitrous oxide (NO) treatment is shown to decrease AKI incidence, which can be caused by hemolysis. Thus, NO treatment itself does not decrease hemolysis but decreases the effect of hemolysis on organ injury [54]. Another medication that is known to reduce the detrimental effects of hemolysis is acetaminophen. However, acetaminophen does not act directly on the by-products of hemolysis, but instead on the lipid peroxidation which can be induced by hemolysis. Acetaminophen reduces lipid peroxidation, thus reducing the incidence of AKI [52, 53].

### Anesthesia

Some studies demonstrate Ca^2+^ as a major factor in RBC membrane homeostasis and deformability. Propofol is known to have free radical scavenging ability and blocking Ca^2+^ channels. This effect is shown in a decrease of PFHb levels in patients given propofol instead of isoflurane 1–2% [55].

### Alternatives to CPB

Recently, there are several alternatives to the use of CPB, to reduce the deleterious effects of CPB. We did a meta-analysis on the common alternatives, Mini Extracorporeal Circulation (MiniECC) and OPCAB. Our analyses show that alternatives to CPB (MiniECC and OPCAB) reduce hemolysis, from PFHb levels. We also found that MiniECC decreased LDH levels. Most of the MiniECC system eliminates the use of cardiotomy suction, which explains the decrease in hemolysis [56–58, 61, 62]. The decreased surface area will minimize priming volume, thus causing less hemodilution. Along with that, the use of Cps in most MiniECC contributes to the decrease of hemolysis in general [57]. Among all studies, only Gunaydin et al. reported finding no significant difference in PFHb formation, with even higher hemolysis in MiniECC [62]. This could be caused by a longer duration in the MiniECC group (98.7 ± 4.2 vs 94.5 ± 3.7) [62]. Nevertheless, MiniECC is still considered superior, in terms of hemolysis, compared to conventional CPB.

Our subgroup analysis also showed that OPCAB causes less hemolysis than CPB. OPCAB eliminates several factors that can cause hemolysis, such as high shear stress, blood-air interface, and blood-non endothelial contact. Therefore, hemolysis in OPCAB is better than in CPB [58, 61, 65].

Other alternatives are Microaxial intracardiac pumps (ICP). We found two studies comparing hemolysis in the use of ICP and CPB. ICP induces more hemolysis in both studies, however, the difference was not significant. Therefore, consideration for to use of ICP over CPB should be assessed based on other factors such as skills and costs [66, 67].

Overall, when available, we suggest opting for alternatives such as MiniECC or OPCAB, which could reduce hemolysis. However, the use of CPB is still unavoidable in complex cardiac surgery.

### Limitations

All meta-analyses have main limitations such as reporting bias, quality assessment, endpoint definitions, and methodological heterogeneity of the included studies.

Reporting bias occurred in full-text publications when the results of the secondary outcome are not positive or not significant. We tried to limit this bias by corresponding with the authors. However, we got limited responses and therefore had to exclude several studies from the meta-analysis and only do narrative synthesis on them. We also assessed the quality of the report using Risk of Bias 2.0 and found seven high-risk biases in selective reporting and one study that reported a difference in the intervention that affected the outcome results. We also found several concerning studies, mostly regarding the randomization process, which were not clearly stated. One study reported a deviation of intervention; however, the deviation did not affect the outcome. One study found significant characteristic differences due to randomization. Another study was not able to do randomization because of the condition of the disease and intervention.

Another major problem is the fact that most RCTs are different. Numerous ECC designs could lead to multiple confounding factors in the outcome results. Time of sampling is also of concern. We generalized the sampling time to be pre-, peri-, and post-operative. However, post-operative timing could range from minutes to days after operations. All those concerns and combining types of cardiac surgery from all ages contribute to the high heterogeneity between studies.

A series of meta-analyses with more specific study parameters (PICOs) needs to be done, to provide better evidence regarding hemolysis and the use of CPB. Also, more in-depth studies are needed, especially research measuring hemolysis and CPB use, or other management related to CPB. Establishing a standardized guideline for the conduct of CPB is also important, to reduce the variance of care, and thereby the confounding factors between studies would be more comparable.

## Conclusions

The limitations of existing data and analysis employed in this meta-analysis and systematic review, we can conclude that hemolysis remains one of the major complications of CPB use. We cannot find a single cause of hemolysis, but CPB duration and the use of cardiotomy suction can be considered the major causes of hemolysis.

There are several ways of incorporating components of the CPB circuit, the conduct of CPB, medications, and even the use of alternatives to reduce hemolysis. Further research needs to be conducted with standardized CPB guidelines to reduce confounding factors and yield better results in identifying factors that are involved in increasing or reducing hemolysis and overall improving the safety of CPB use.

### Supplementary Information

**Additional file 1.** Supplementary Tables and Figures.

## Data Availability

The datasets used and/or analysed during the current study are available from the corresponding author on reasonable request.

## Abbreviations

CPB: Cardiopulmonary bypass
PFHb: Plasma free hemoglobin
hp: Haptoglobin
AKI: Acute kidney injury
CENTRAL: Cochrane central register of controlled trials
RoB: Risk of bias
MD: Mean difference
MiniECC: Mini extracorporeal circulation
OPCAB: Off-pump coronary artery bypass
ICP: Intracardiac pump
CP: Centrifugal pump
RP: Roller pump
VAVD: Vacuum-assisted venous drainage
GAVD: Gravity-assisted venous drainage
HFMO: Hollow-fiber membrane oxygenator
MO: Membrane oxygenator
BO: Bubble oxygenator
HO: Hybrid oxygenator
CS: Cardiotomy suction
SS: SmartSuction
CATS: Continous auto-transfusion system
SC: SmartCannula
NO: Nitric oxide
PEO: Plasma electrolytic oxidation
Pc: Phosphorylcholine
PMEA: Poly-2-methoxyethylacrylate
TCC: Terminal complement complex
PTX: Pentoxifylline
ECC: Extracorporeal circulation
