# Supplementary material for: Hemolysis and cardiopulmonary bypass: meta-analysis and systematic review of contributing factors
Source: J Cardiothorac Surg. 2023 Oct 13;18:291. doi: 10.1186/s13019-023-02406-y (PMC10571250; doi:10.1186/s13019-023-02406-y)
Supplement: Supplementary file 1 — Additional file 1. Supplementary Tables and Figures. [file 13019_2023_2406_MOESM1_ESM.docx]

**SUPPLEMENTAL MATERIALS**

**S1.** Detailed Search Strategy,

**Cochrane CENTRAL**

| ID | Search | Hits |
| --- | --- | --- |
| #1 | MeSH descriptor: [Cardiac Surgical Procedures] explode all trees | 12967 |
| #2 | MeSH descriptor: [Heart Defects, Congenital] explode all trees | 2255 |
| #3 | (cardiac surger*):ti,ab,kw OR (heart surger*):ti,ab,kw OR (coronary artery bypass*):ti,ab,kw OR (CABG):ti,ab,kw OR (Valve surger*):ti,ab,kw | 24978 |
| #4 | ("valve replacement"):ti,ab,kw OR (valve-plasty):ti,ab,kw OR (Pediatric Heart Surger*):ti,ab,kw OR (Congenital Heart Surger*):ti,ab,kw OR (Coronary | 7301 |
| #5 | **#1 OR #2 OR #3 OR #4** | **34413** |
| #6 | MeSH descriptor: [Cardiopulmonary Bypass] explode all trees | 2737 |
| #7 | MeSH descriptor: [Heart-Lung Machine] explode all trees | 37 |
| #8 | ("cardiopulmonary bypass"):ti,ab,kw OR (CPB):ti,ab,kw OR (Bypass machine):ti,ab,kw OR ("heart lung machine"):ti,ab,kw OR (heart bypass):ti,ab,kw | 12713 |
| #9 | (heart lung bypass):ti,ab,kw OR (On-pump cardiac surgery):ti,ab,kw OR (On-pump heart surgery):ti,ab,kw | 911 |
| #10 | **#6 OR #7 OR #8 OR #9 OR** | **12730** |
| #11 | **#5 AND #10** | **11305** |
| #12 | ("randomized-controlled trial"):pt OR ("controlled clinical trial"):pt OR (randomized):ti,ab,kw OR (placebo):ti,ab,kw | 1245881 |
| #13 | MeSH descriptor: [] explode all trees and with qualifier(s): [drug therapy - DT] | 204370 |
| #14 | (randomly):ti,ab,kw OR (trial):ti,ab,kw OR (groups):ti,ab,kw | 1086557 |
| #15 | MeSH descriptor: [Animals] explode all trees | 601438 |
| #16 | MeSH descriptor: [Humans] explode all trees | 601378 |
| #17 | #15 NOT #16 | 60 |
| #18 | **#12 OR #13 OR #14 NOT #17** | **1386573** |
| #19 | MeSH descriptor: [Hemolysis] explode all trees | 230 |
| #20 | (Haemolysis):ti,ab,kw OR (Hemolysis):ti,ab,kw | 1249 |
| #21 | (Plasma Free Hemoglobin):ti,ab,kw OR (Lactate Dehydrogenase):ti,ab,kw OR (Haptoglobin):ti,ab,kw OR (LDH):ti,ab,kw OR (PFHb):ti,ab,kw | 4223 |
| #22 | (microRNA):ti,ab,kw | 628 |
| #23 | **#19 OR #20 OR #21 OR #22** | **5810** |
| #24 | **#11 AND #18 AND #23** | **213** |
| #25 | Then limit the year: 1990-2021, Clinical Trials tab | **205** |

**PubMed Database**

| ID | Search | Hits |
| --- | --- | --- |
| #1 | MeSH descriptor: [Cardiac Surgical Procedures] explode all trees |  |
| #2 | MeSH descriptor: [Heart Defects, Congenital] explode all trees |  |
| #3 | (cardiac surger*):ti,ab,kw OR (heart surger*):ti,ab,kw OR (coronary artery bypass*):ti,ab,kw OR (CABG):ti,ab,kw OR (Valve surger*):ti,ab,kw |  |
| #4 | ("valve replacement"):ti,ab,kw OR (valve-plasty):ti,ab,kw OR (Pediatric Heart Surger*):ti,ab,kw OR (Congenital Heart Surger*):ti,ab,kw OR (Coronary |  |
| #5 | **#1 OR #2 OR #3 OR #4** | **406.711** |
| #6 | MeSH descriptor: [Cardiopulmonary Bypass] explode all trees |  |
| #7 | MeSH descriptor: [Heart-Lung Machine] explode all trees |  |
| #8 | ("cardiopulmonary bypass"):ti,ab,kw OR (CPB):ti,ab,kw OR (Bypass machine):ti,ab,kw OR ("heart lung machine"):ti,ab,kw OR (heart bypass):ti,ab,kw |  |
| #9 | (heart lung bypass):ti,ab,kw OR (On-pump cardiac surgery):ti,ab,kw OR (On-pump heart surgery):ti,ab,kw |  |
| #10 | **#6 OR #7 OR #8 OR #9 OR** | **44.463** |
| #11 | **#5 AND #10** | **28.440** |
| #12 | MeSH descriptor: [Hemolysis] explode all trees | 230 |
| #13 | (Haemolysis):ti,ab,kw OR (Hemolysis):ti,ab,kw | 1249 |
| #14 | (Plasma Free Hemoglobin):ti,ab,kw OR (Lactate Dehydrogenase):ti,ab,kw OR (Haptoglobin):ti,ab,kw OR (LDH):ti,ab,kw OR (PFHb):ti,ab,kw | 4223 |
| #15 | (microRNA):ti,ab,kw | 628 |
| #16 | **#12 OR #13 OR #14 OR #15** | **165.312** |
| #17 | **#11 AND #16** | **164** |
|  | Filter : 1990 - 2021 | **154** |
|  | Filter : RCT and Clinical Trial, 1990 - 2021 | **93** |

**S2.** Study Characteristics Table

| **Author, Year** | **No. of Patients** | **Types of Surgery** | **Intervention** | **Control Group** | **Hemolysis Parameters** | **Timing of sampling** |
| --- | --- | --- | --- | --- | --- | --- |
| Hansbro 1999 | 60 | CABG | Centrifugal Pump / Dynamic Roller Pump | Roller Pump | PFHb (mg/dL) | Perioperative |
| Valeri 2006 | 14 | CABG | Centrifugal Pump | Roller Pump | PFHb (mg/dL)  LDH (U/L) | Perioperative |
| Murakami 1997 | 30 | CABG | Centrifugal Pump | Roller Pump | PFHb (mg/dL)  Hp (mg/dL)  LDH (IU/L) | Perioperative |
| Passaroni 2018 | 60 | CABG | Centrifugal Pump | Roller Pump | Hp (mg/dL)  LDH (mg/dL) | Intraoperative,  Postoperative |
| Fransen 2005 | 22 | CABG | Centrifugal Pump | Roller Pump | PFHb (µmol/L)  Hp (g/L) | Intraoperative,  Postoperative |
| Morgan 1998 | 42 | Congenital Heart Surgery | Centrifugal Pump | Roller Pump | PFHb (mg/dL)  Hp (g/L) | Intraoperative,  Postoperative |
| Nishinaka 1996 | 24 | CABG | Centrifugal Pump (Impeller) | Roller Pump (Model PSa) | PFHb (mg/dL)  Index of Hemolysis | Intraoperative,  Postoperative |
| Keyser 2011 | 240 | CABG | Centrifugal Pump | Roller Pump | LDH (U/L) | Intraoperative,  Postoperative |
| Jakob 1991 | 50 | CABG | Centrifugal Pump | Roller Pump (Stockert) | PFHb (mg/L) | Intraoperative,  Postoperative |
| Andersen 2003 | 34 | CABG | Centrifugal Pump | Roller Pump | PFHb (mg/L) | Intraoperative,  Postoperative |
| Wheeldon 1990 | 16 | CABG | Vortex Pump (Centrifugal) | Roller Pump | PFHb (mg/L) | Perioperative |
| Paparella 2004 | 20 | CABG | Revolution Centrifugal Pump | Bio-Pump Centrifugal Pump | PFHb (g/dL) | Perioperative |
| Ohtsubo 1996 | 20 | Aortic Valve Surgery 2  ASD closure 1  CABG 17 | Nikkiso Centrifugal Pump | BioMedicus Centrifugal Pump | PFHb (mg/dL) | Perioperative |
| Benedetti 1990 | 26 | CABG | Bubble oxygenator (BO), Membrane oxygenator (MO) 'hybrid' oxygenator (HO), and hollow fibre oxygenator (HFO) | - | PFHb (mg/100mL) | Perioperative |
| Simons 2010 | 33 | CABG | HF oxygenator with a relatively low hydraulic resistance | HF oxygenator with a relatively high hydraulic resistance | Normalized Index of Hemolysis | Intraoperative |
| Stammers 1998 | 26 | CABG | Hollow Fiber Membrane Oxygenator | Membrane Oxygenators | PFHb (mg/dL) | Preoperative, Intraoperative |
| Chukwuemeka 2000 | 20 | CABG | Hollow Fiber Oxygenator (HF-5700), Polymer heat exchanger | Hollow Fiber Oxygenator (HF-6700), Stainless steel heat exchanger | Haptoglobin (mg/dL) | Preoperative, Intraoperative |
| Jegger 2006 | 13 | 4 CABG  5 Valve surgery  2 CABG + Valve  2 others | SmartCanula | Control | PFHb (mg/L)  LDH (U/L) | N/A |
| Jegger 2007 | 56 | 25 CABG, 19 Valve surgery, 5 combined surgery, 13 others | SmartSuction | Cardiotomy Suction | PFHb (mg/L)  LDH (U/L) | Perioperative |
| Tanaka 2003 | 19 | Valve Surgery | Closed Circuit ECC  (Separated Reservoir) | Open Circuit  (Combined Reservoir) | PFHb (mg/dL) | Perioperative |
| Gunaydin 2010 | 48 | CABG | Closed Circuit + Hyaluronan Coated / Closed Circuit + Hyaluronan Uncoated / Open Circuit + Hyaluronan Coated | Open and Uncoated Circuit | PFHb (µmol/L) | Perioperative |
| Pierangeli 2001 | 21 | CABG | Separation of suctioned blood | Suction blood returned to venous reservoir | PFHb (mg/dL) | Intraoperative, Postoperative |
| Nasso 2011 | 150 | 66 CABG, 21 AVR, 18 MVR, 17 Mitral Valve Repair, 9 Ascending Aortic Replacement, 19 CABG + Valve Replacement | EVADO group | Conventional CPB | PFHb (%variation)  Haptoglobin (%variation) | Perioperative |
| Nakajima 1995 | 24 | CABG | Heparin Coated Circuit | Uncoated Circuit (Control) | PFHb (mg/dL) | Perioperative |
| Sellevold 1994 | 20 | CABG | Heparin Coated Circuit | Uncoated Circuit (Control) | PFHb (g/L) | Perioperative |
| Moen 1996 | 40 | CABG | Heparin Coated Oxygenators / Centrifugal Pump | Uncoated Oxygenators/ Roller Pump | PFHb (g/dL) | Perioperative |
| Pappalardo 2006 | 44 | N/A | Phosphorylcholine Coated Oxygenators | Uncoated Oxygenators | PFHb (mg/L) | Perioperative |
| De Somer 2002 | 20 | CABG | Phosphorylcholine coating | Uncoated Circuit (Control) | PFHb, Hp | Perioperative |
| De Somer 2001 | 30 | CABG | Suction blood discarded + Phosphorylcholine coating | Suction Blood Discarded, Uncoated | PFHb (mg/100mL or mg/dL),  Hp (%difference from baseline) | Intraoperative  Postoperative |
| Thiara 2010 | 30 | 3 CABG, 27 Combined | Phosphorylcholine coating | Heparin Coating | LDH (U/L) | Intraoperative, Postoperative |
| Thiara 2011 | 30 | 1 CABG, 29 combined | Phosphorylcholine coating | PMEA Coated | LDH (U/mL) | Intraoperative, Postoperative |
| Jacobs 2011 | 30 | CABG | Heparin + Phosphorylcholine Coating | Phosphorylcholine Coating | Haptoglobin (g/L)  Hemolytic Index (mg/dL) | Intraoperative, Postoperative |
| Bevilacqua 2002 | 72 | CABG | VAVD | Gravitational Venous Drainage | PFHb (mg/dL) | Intraoperative,  Postoperative |
| Hayashi 2001 | 60 | Valve Surgery 28  CABG 27  Congenital 5 | VAVD | Siphone Dependent VD (Gravity) | PFHb (mg/dL)  Hp (Reduction Ratio, %/min) | Perioperative |
| Goksedef 2011 | 162 | CABG | VAVD at 40 mmHg and VAVD at 80 mmHg | Gravity Siphon VD | PFHb (mg/dL)  Hp (mg/dL)  LDH (IU/L) | Preoperative,  Postoperative |
| Minami 1990 | 30 | CABG | Pulsatile | Non-pulsatile | PFHb (mg/dL) | Preoperative,  Postoperative |
| Zhao 2011 | 40 | Congenital Heart Surgery | Pulsatile | Non-pulsatile | PFHb (mg/L) | Perioperative |
| Kocakulak 2004 | 40 | N/A | Pulsatile | Non-pulsatile | PFHb (mg/dL) | Intraoperative,  Postoperative |
| Song 1997 | 70 | 25 MVR, 11 AVR+MVR, 9 TOF, 11 ASD, 10 VSD, 4 DORV | Pulsatile | Non-pulsatile | PFHb (mg/L) | Perioperative |
| Zhao 2009 | 24 | Congenital Heart Surgery (ToF) | Pulsatile | Non-pulsatile | PFHb (mg/L) | Perioperative |
| Skrabal 2006 | 35 | CABG | Retransfusion of suctioned blood | retainment of suctioned blood | PFHb (mg/L) | Preoperative,  Postopertative |
| de Haan 1995 | 40 | CABG | retransfusion of suctioned blood | retainment of suctioned blood | PFHb (µg/mL) | Intraoperative |
| Walpoth 1999 | 20 | CABG | Retransfusion with CATS | Retransfusion of unprocessed blood | PFHb (mg/dL) | Intraoperative,  Postoperative |
| Gleason 2019 | 38 | N/A | CytoSorb as an adjunct to standard of care (TREATMENT). | standard of care (CONTROL) | PFHb (mg/dL) | Intraoperative,  Postoperative |
| Boey 1993 | 40 | CABG | Plateletpheresis | Control | Haptoglobin | Perioperative |
| Rinne 1996 | 100 | CABG | blood cardioplegia (BCP) | crystalloid cardioplegia (CCP). | PFHb (mg/L) | Intraoperative,  Postoperative |
| Barbu 2020 | 80 | 27 CABG  26 Valve surgery  14 Valve + CABG  13 Others | Dextran-based prime | A crystalloid prime containing Ringer acetate and mannitol. | PFHb (g/L) | Intraoperative,  Postoperative |
| Shihai 2004 | 40 | Congenital Heart Surgery | propofol group | Control (1-2% Isoflurane) | PFHb (mg/L) | Perioperative |
| Golbasi 2006 | 50 | CABG | Pentoxifylline | Placebo | PFHb (mg/dL)  Hp (mg/dL) | Perioperative |
| Nikolay 2020 | 96 | 41 CABG, 22 Valve Replacement, 7 CABG, MABG + SVR, 26 CABG, MABG + Valve Replacement | NO treatment | Placebo | PFHb (g/L) | Intraoperative, Postoperative |
| Simpson 2014 | 30 | Congenital Heart Surgery | Acetaminophen Injection | Placebo | PFHb (mg/dL), Hp (mg/dL) | Perioperative |
| Billings 2015 | 60 | 48 Valve Surgery, 1 CABG, 7 Combined surgery (CABG+Valve), 3 Congenital Surgery, 1 Other | Acetaminophen Injection | Placebo | PFHb (mg/dL), Hp (mg/dL) | Perioperative |
| Kiessling 2018 | 72 | CABG | MiniECC/ Closed ECC | Open ECC (standard CPB) | PFHb (mg/dL) | Intraoperative,  Postoperative |
| Gunaydin 2009 | 40 | CABG | MiniECC | standard CPB | PFHb (µmol/L) | Intraoperative,  Postoperative |
| Beghi 2006 | 60 | CABG | MiniECC | standard CPB | PFHb (g/L) | Perioperative |
| Deininger 2015 | 120 | CABG | MiniECC / OPCAB | standard CPB | PFHb (mg/dL) | Intraoperative,  Postoperative |
| Liu 2011 | 40 | CABG | MiniECC | standard CPB | PFHb (mg/L) | Intraoperative,  Postoperative |
| Anastasiadis 2010 | 99 | CABG | MiniECC | standard CPB | LDH (mg/dL) | Perioperative |
| Ng 2014 | 78 | CABG | MiniECC | standard CPB | LDH (U/L) | Intraoperative,  Postoperative |
| Chew 2015 | 67 | CABG | MiniECC / OPCAB | standard CPB | LDH (U/L) | Intraoperative,  Postoperative |
| Wippermann 2005 | 30 | CABG | CCECC (MiniECC) / OPCAB | standard CPB | PFHb (µmol/L) | Perioperative |
| Autschbach 2001 | 80 | CABG | A new device for in heart biventricular intracorporeal circulation was applied | Conventional CPB | PFHb (mg/dL) | Intraoperative,  Postoperative |
| Caputo 2002 | 60 | CABG | off pump group, RVAD group | Conventional CPB | PFHb (mg/dL) | Perioperative |
| Meyns 2002 | 199 | CABG | Intracardiac Microaxial Pump | Conventional CPB | PFHb (mg/dL) | Perioperative |

**S3.** Revised Cochrane risk of bias tool for randomized trials (RoB 2.0)

| Study ID | D1 | D2 | D3 | D4 | D5 | Overall |
| --- | --- | --- | --- | --- | --- | --- |
| Hansbro 1999 | **!** 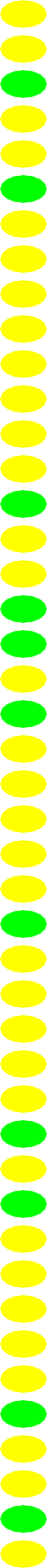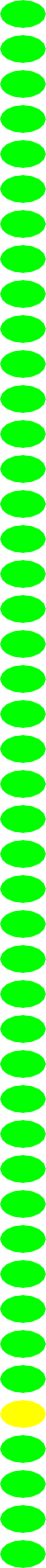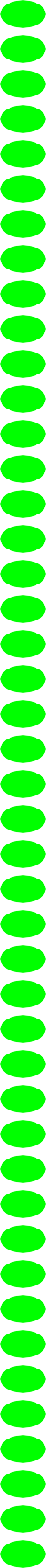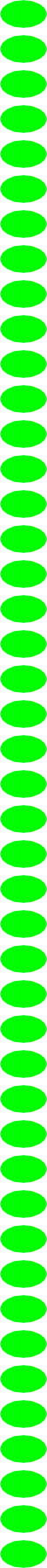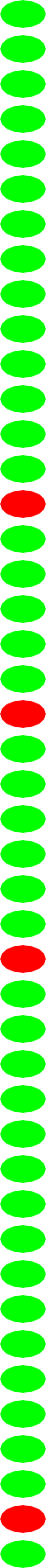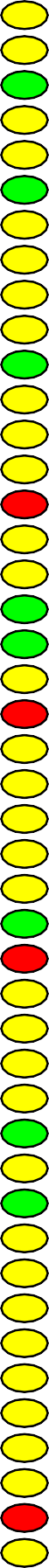 | **+** | **+** | **+** | **+** | **!** |
| Valeri 2006 | **!** | **+** | **+** | **+** | **+** | **!** |
| Murakami 1997 | **+** | **+** | **+** | **+** | **+** | **+** |
| Passaroni 2018 | **!** | **+** | **+** | **+** | **+** | **!** |
| Fransen 2005 | **!** | **+** | **+** | **+** | **+** | **!** |
| Morgan 1998 | **+** | **+** | **+** | **+** | **+** | **+** |
| Nishinaka 1996 | **!** | **+** | **+** | **+** | **+** | **!** |
| Keyser 2011 | **!** | **+** | **+** | **+** | **+** | **!** |
| Jakob 1991 | **!** | **+** | **+** | **+** | **+** | **!** |
| Andersen 2003 | **!** | **+** | **+** | **+** | **+** | **!** |
| Wheeldon 1990 | **!** | **+** | **+** | **+** | **+** | **+** |
| Paparella 2004 | **!** | **+** | **+** | **+** | **+** | **!** |
| Ohtsubo 1996 | **!** | **+** | **+** | **+** | **+** | **!** |
| Benedetti 1990 | **!** | **+** | **+** | **+** | **+** | **!** |
| Simons 2010 | **+** | **+** | **+** | **+** | **-** | **-** |
| Stammers 1998 | **!** | **+** | **+** | **+** | **+** | **!** |
| Chukwuemeka 2000 | **!** | **+** | **+** | **+** | **+** | **!** |
| Jegger 2006 | **+** | **+** | **+** | **+** | **+** | **+** |
| Jegger 2007 | **+** | **+** | **+** | **+** | **+** | **+** |
| Tanaka 2003 | **!** | **+** | **+** | **+** | **+** | **!** |
| Gunaydin 2010 | **+** | **+** | **+** | **+** | **-** | **-** |
| Pierangeli 2001 | **!** | **+** | **+** | **+** | **+** | **!** |
| Nasso 2011 | **!** | **+** | **+** | **+** | **+** | **!** |
| Nakajima 1995 | **!** | **+** | **+** | **+** | **+** | **!** |
| Sellevold 1994 | **!** | **+** | **+** | **+** | **+** | **!** |
| Moen 1996 | **!** | **+** | **+** | **+** | **+** | **!** |
| Pappalardo 2006 | **+** | **+** | **+** | **+** | **+** | **+** |
| De Somer 2002 | **!** | **+** | **+** | **+** | **-** | **-** |
| De Somer 2001 | **!** | **+** | **+** | **+** | **+** | **!** |
| Thiara 2010 | **!** | **+** | **+** | **+** | **+** | **!** |
| Thiara 2011 | **!** | **+** | **+** | **+** | **+** | **!** |
| Jacobs 2011 | **!** | **+** | **+** | **+** | **+** | **!** |
| Bevilacqua 2002 | **+** | **+** | **+** | **+** | **+** | **+** |
| Hayashi 2001 | **!** | **+** | **+** | **+** | **+** | **!** |
| Goksedef 2011 | **+** | **+** | **+** | **+** | **+** | **+** |
| Minami 1990 | **!** | **+** | **+** | **+** | **+** | **!** |
| Zhao 2011 | **!** | **+** | **+** | **+** | **+** | **!** |
| Kocakulak 2004 | **!** | **+** | **+** | **+** | **+** | **!** |
| Song 1997 | **!** | **+** | **+** | **+** | **+** | **!** |
| Zhao 2009 | **!** | **+** | **+** | **+** | **+** | **!** |
| Skrabal 2006 | **+** | **!** | **+** | **+** | **+** | **!** |
| de Haan 1995 | **!** | **+** | **+** | **+** | **+** | **!** |
| Walpoth 1999 | **!** | **+** | **+** | **+** | **+** | **!** |
| Gleason 2019 | **+** | **+** | **+** | **+** | **-** | **-** |
| Boey 1993 | **!** | **+** | **+** | **+** | **+** | **!** |

| Rinne 1996 | **!** | **+** | **+** | **+** | **+** 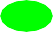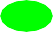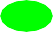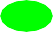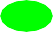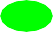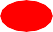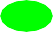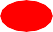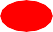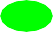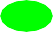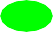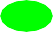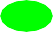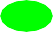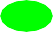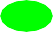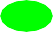 | **!** 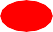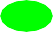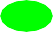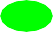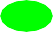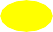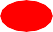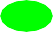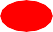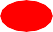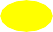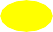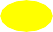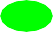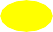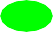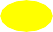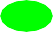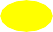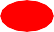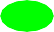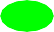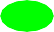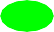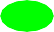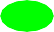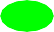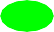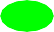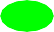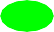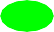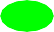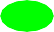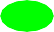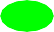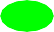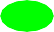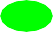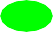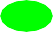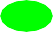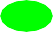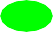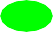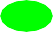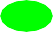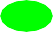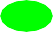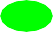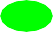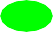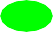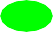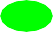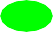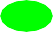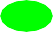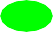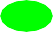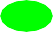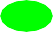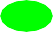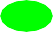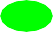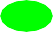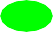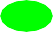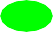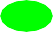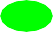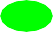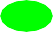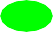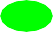 |
| --- | --- | --- | --- | --- | --- | --- |
| Barbu 2020 | **+** | **+** | **+** | **+** | **+** | **+** |
| Shihai 2004 | **!** | **+** | **+** | **+** | **+** | **!** |
| Golbasi 2006 | **!** | **+** | **+** | **+** | **+** | **!** |
| Nikolay 2020 | **+** | **+** | **+** | **+** | **+** | **+** |
| Simpson 2014 | **+** | **+** | **+** | **-** | **+** | **-** |
| Billings 2015 | **+** | **+** | **+** | **+** | **+** | **+** |
| Kiessling 2018 | **!** | **+** | **+** | **+** | **-** | **-** |
| Gunaydin 2009 | **+** | **+** | **+** | **+** | **-** | **-** |
| Beghi 2006 | **+** | **+** | **+** | **+** | **+** | **+** |
| Deininger 2015 | **!** | **+** | **+** | **+** | **-** | **-** |
| Liu 2011 | **!** | **+** | **+** | **+** | **+** | **!** |
| Anastasiadis 2010 | **+** | **+** | **+** | **+** | **+** | **+** |
| Ng 2014 | **+** | **+** | **+** | **+** | **+** | **+** |
| Chew 2015 | **+** | **+** | **+** | **+** | **+** | **+** |
| Wippermann 2005 | **!** | **+** | **+** | **+** | **+** | **!** |
| Autschbach 2001 | **!** | **+** | **+** | **+** | **+** | **!** |
| Caputo 2002 | **+** | **+** | **+** | **+** | **+** | **+** |
| Meyns 2002 | **!** | **+** | **+** | **+** | **+** | **!** |

**S4.** Narrative Synthesis Table

| **Study** | **PFHb** | **Hp** | **LDH** | **IH** |
| --- | --- | --- | --- | --- |
| Simons 2010 | NR | NR | NR | LRO: 0.088 ± 0.074  HRO: 0.104 ± 0.088 |
| Chukwuemeka 2000 | NR | HF6700: 87 ± 58 mg/dL  HF5700: 106 ± 81 mg/dL  P value NS (number NR) | NR | NR |
| Paparella 2004 | PFHb in graph, Graph extraction N/A | NR | NR | NR |
| Ohtsubo 1996 | PFHb in graph | NR | NR | NR |
| Jakob 1991 | PFHb in graph, Graph extraction N/A  p value < 0.05 | NR | NR | NR |
| Andersen 2003 | PFHb in graph, Graph extraction N/A | NR | NR | NR |
| Wheeldon 1990 | PFHb in graph, Graph extraction N/A | NR | NR | NR |
| Bevilacqua 2002 | PFHb in graph, graph extraction N/A  p value NR | NR | NR | NR |
| Jegger 2006 | PFHb  CTRL : 549 ± 271  SC : 568 ± 142  p value NR | NR | LDH  CTRL : 354 ± 116  SC : 335 ± 73  p value NR |  |
| Jegger 2007 | PFHb (peri-CPB)  SS : 351 ± 176 mg/L  CTRL : 486 ± 204 mg/L  (p < 0.05)  PFHb (post-CPB)  SS : 460 ± 254 mg/L  CTRL : 549 ± 271 mg/L  (p < 0.05) | NR | LDH (peri-CPB)  SS : 207 ± 83 U/L  CTRL : 275 ± 100 U/L  (p < 0.05)  LDH (post-CPB)  SS : 275 ± 89 U/L  CTRL : 354 ± 116 U/L  (p < 0.01) | NR |
| Moen 1996 | PFHb in Graph, Extraction from Graph N/A | NR | NR | NR |
| Jacobs 2011 | NR | PC : 1.38±0.54 mg/dL  XPC : 1.56±0.52 mg/dL  p value NR | NR | NR |
| De Somer 2002 | No Tabular Data, Graph N/A | NR | NR | NR |
| Barbu 2020 | PFHb (2h post CPB)  0.18±0.11g/L  0.41±0.33 g/L  p=0.001 | NR | NR | NR |
| Rinne 1996 | BCP : 220.2 ± 148.9 mg/L  CCP : 207.5 ± 107.4 mg/L  p = 0.084 | NR | NR | NR |
| Nasso 2011 | CPB : 24.45%variation  EVADO : 11.46%variation  p < 0.0001 | CPB : -17.26%  EVADO : -11.85%  p < 0.0001 | NR | NR |
| Walpoth 1999 | Control : 45±12 mg/dL  CATS : 7±2 mg/dL  p value (<0.05) | NR | NR | NR |
| Gleason 2019 | Data unclear | NR | NR | NR |
| Boey 1993 | NR | Haptoglobin <0.3 g/dL  PRP : 8/19  Control : 0/20  p < 0.005 | NR | NR |
| Golbasi 2006 | PFHb in Graph  p < 0.05 | Haptoglobin in Graph  p < 0.05 | NR | NR |
| Kamenschikov 2020 | PFHb in Graph  p = 0.98 | NR | NR | NR |
| Simpson 2014 | PFHb in Graph,  p = 0.07 | Haptoglobin in Graph  p = 0.29 | NR | NR |
| Billings 2015 | PFHb in Graph  p = 0.52 | Haptoglobin in Graph  p = 0.97 | NR | NR |
| Shihai 2004 | PFHb :  C : 125.0 ± 44.2  P : 83.1 ± 28.4  p <0.01 | NR | NR | NR |
| Autschbach 2001 | Reported in graph format, Graph Extraction N/A | NR | NR | NR |
| Meyns 2002 | Reported in graph format, Graph Extraction N/A | NR | NR | NR |
| Kiessling 2018 | MiniECC : 7.54  OpenECC : 8.09  ClosedECC : 7.19  p value NR  SD NR | NR | NR | NR |
| Anastasiadis 2010 | NR | NR | LDH (ICU arrival)  MiniECC: 633.73±275.05  ConventionalECC: 951.95±564.68  p < 0.001 | NR |

**S5.** Forest plot of published studies comparing lactate dehydrogenase (LDH) between centrifugal pumps and roller pumps using random effects analysis. Data presented as Mean Difference (MD) with 95% Confidence Interval (CI).

**S6.** Forest plot of published studies comparing plasma free hemoglobin (PFHb) between hollow fibre membrane oxygenators (HFMO) and membrane oxygenators (MO) using random effects analysis. Data presented as Mean Difference (MD) with 95% Confidence Interval (CI).

**S7.** Forest plot of published studies comparing plasma free hemoglobin (PFHb) of coatings using random effect analysis. Subgroup analyses were done for heparin coating and phosphorylcholine coating using random effect analysis. Data presented as Mean Difference (MD) with 95% Confidence Interval (CI).

**S8.** Forest plot of published studies comparing lactate dehydrogenase (LDH) between phosphorylcholine (Pc) and non-phosphorylcholine coating using random effects analysis. Data presented as Mean Difference (MD) with 95% Confidence Interval (CI).
